# Rapamycin (mTORC1 inhibitor) reduces the production of lactate and 2-hydroxyglutarate oncometabolites in IDH1 mutant fibrosarcoma cells

**DOI:** 10.1186/s13046-017-0544-y

**Published:** 2017-06-02

**Authors:** Zoltán Hujber, Gábor Petővári, Norbert Szoboszlai, Titanilla Dankó, Noémi Nagy, Csilla Kriston, Ildikó Krencz, Sándor Paku, Olivér Ozohanics, László Drahos, András Jeney, Anna Sebestyén

**Affiliations:** 1 1st Department of Pathology and Experimental Cancer Research, Semmelweis University, Üllői út 26, Budapest, 1085 Hungary; 2 Laboratory of Environmental Chemistry and Bioanalytics, Department of Analytical Chemistry, Institute of Chemistry, Eötvös Loránd University, 1518 Budapest, Hungary; 3 Tumor Progression Research Group of Joint Research Organization of Hungarian Academy of Sciences and Semmelweis University, Budapest, Hungary; 4 Hungarian Academy of Sciences, Research Centre for Natural Sciences, MS Proteomics Research Group, 1117 Budapest, Hungary

**Keywords:** Rapamycin, mTOR, Oncometabolite, 2-hydroxyglutarate, Lactate, Tumour metabolism

## Abstract

**Background:**

Multiple studies concluded that oncometabolites (e.g. D-2-hydroxyglutarate (2-HG) related to mutant isocitrate dehydrogenase 1/2 (*IDH1/2*) and lactate) have tumour promoting potential. Regulatory mechanisms implicated in the maintenance of oncometabolite production have great interest. mTOR (mammalian target of rapamycin) orchestrates different pathways, influences cellular growth and metabolism. Considering hyperactivation of mTOR in several malignancies, the question has been addressed whether mTOR operates through controlling of oncometabolite accumulation in metabolic reprogramming.

**Methods:**

HT-1080 cells – carrying originally endogenous IDH1 mutation – were used in vitro and in vivo. Anti-tumour effects of rapamycin were studied using different assays. The main sources and productions of the oncometabolites (2-HG and lactate) were analysed by ^13^C-labeled substrates. Alterations at protein and metabolite levels were followed by Western blot, flow cytometry, immunohistochemistry and liquid chromatography mass spectrometry using rapamycin, PP242 and different glutaminase inhibitors, as well.

**Results:**

Rapamycin (mTORC1 inhibitor) inhibited proliferation, migration and altered the metabolic activity of *IDH1* mutant HT-1080 cells. Rapamycin reduced the level of 2-HG sourced mainly from glutamine and glucose derived lactate which correlated to the decreased incorporation of ^13^C atoms from ^13^C-substrates. Additionally, decreased expressions of lactate dehydrogenase A and glutaminase were also observed both in vitro and in vivo.

**Conclusions:**

Considering the role of lactate and 2-HG in regulatory network and in metabolic symbiosis it could be assumed that mTOR inhibitors have additional effects besides their anti-proliferative effects in tumours with glycolytic phenotype, especially in case of *IDH1* mutation (e.g. acute myeloid leukemias, gliomas, chondrosarcomas). Based on our new results, we suggest targeting mTOR activity depending on the metabolic and besides molecular genetic phenotype of tumours to increase the success of therapies.

**Electronic supplementary material:**

The online version of this article (doi:10.1186/s13046-017-0544-y) contains supplementary material, which is available to authorized users.

## Background

Mammalian target of rapamycin (mTOR) kinase represents an important regulator of cellular metabolism besides of its effects on cellular growth, protein synthesis, proliferation and survival. mTOR senses energy supply, nutrients, growth factors and other conditions in tumour microenvironment (e.g. oxygen, stress) at the crossroad of cellular signalling networks [1–3]. Available energy and nutrients besides appropriate growth factors allow mTOR activity which supports anabolic pathways, however, the absence of the above mentioned factors leads to the inactivation of mTOR and inhibits the anabolism and induces the cellular catabolism, such as autophagy. mTOR complexes orchestrate different pathways and based on their input influence cellular growth and metabolic programs. mTOR kinase exists in two structurally and functionally different protein complexes: rapamycin sensitive mTORC1 and rapamycin-resistant mTORC2 (however, mTORC2 could be rapamycin sensitive during a long-term treatment) [4, 5]. The multiple regulatory functions of mTOR complexes are achieved through different downstream targets. Translation, ribosome and mitochondrial biogenesis, autophagy, lipid biosynthesis are controlled by mTORC1, which phosphorylates e.g. p70S6K and 4-EBP1 and consequently other targets such as S6 which is a well-known marker of mTORC1 activity at protein level. mTORC2 phosphorylates e.g. Akt (at S473), SGK1 and PKCα and enhances survival and cytoskeletal reorganization [3]. Data about other functions of mTORC2 are emerging e.g. a potential metabolic regulatory role of mTORC2 is also in the centre of interest in recent studies.

The failures in PI3K/Akt/mTOR signalling pathway and hyperactivated mTOR kinase have been described in many tumours. In fact, a correlation between elevated mTOR activity – especially with potential high mTORC2 activity (mTORC2 complex related Rictor overexpression) – and poor clinical prognosis of different malignancies has been previously described in our and other studies [6–8]. Rapamycin (mTORC1 inhibitor) and its analogues (rapalogs; e.g. temsirolimus, everolimus) have been approved by FDA (Food and Drug Administration) and EMA (European Medicines Agency) in certain lymphomas, renal cell carcinomas, pancreatic and breast cancers; rapalogs and newly developing inhibitor phase studies are ongoing in many other malignancies.

The regulatory role of mTOR activity in the metabolic reprogramming has been highlighted in recent years. mTORC1 complex activity is necessary for appropriate glycolysis, glutamine consumption, pentose-phosphate pathway, lipid and nucleotide synthesis; moreover, it downregulates autophagy [9–11]. Through regulating these mechanisms, mTOR activity can help the adaptation to altered energy-, nutrient supplies and microenvironment in metabolic reprogramming [12–14].

Lactate production is a result of Warburg effect in different tumours [10, 15]. Lactate can be regarded as an oncometabolite – oncometabolites are defined as small-molecule components of normal metabolism of which accumulation causes signalling dysregulation to establish a milieu that initiates carcinogenesis – based on emerging data. Lactate contributes to many effects which help the survival and clonal evolution of resistant tumour cells in tissue microenvironment [16–18]. The tumour microenvironment is a complex network of extracellular matrix molecules, soluble factors, non-tumorous cells, including stromal (e.g. cancer associated fibroblasts and endothelial cells, adipocytes) and immunoregulatory cells. Among the soluble factors present in the microenvironment, lactate production has particular importance through its effects not only in cancer, but also, in neighbouring cells. In vivo lactic acid contributes to acidosis, which – besides triggering pain in cancer patients – has more important signals for tumour angiogenesis and tumour immunosuppression, as well [19]. In addition, the decreasing pH may contribute to the metastasis formation (e.g. through MMP activation, matrix remodelling) of some cancers [19].

Moreover, tumour and stromal cells can utilize lactate for energy production depending on the oxygen concentration in metabolic symbiosis, as well [20, 21]. Certain dysfunctioning metabolic enzymes – e.g. mutated FH, SDH or IDH (mitochondrial IDH2 or cytoplasmic IDH1) – promote the accumulation of other oncometabolites such as fumarate, succinate and D-2-hydroxyglutarate [17, 22]. 2-hydroxyglutarate could exist as L- or D-enantiomers. Low level of L-2-HG could be produced independently from mutant IDH activity in certain cells at hypoxic conditions (e.g. malate dehydrogenase). However, D-2-HG (2-HG) is generated from α-KG by the mIDH and the level of this oncometabolite is highly elevated in mIDH bearing malignant cells [23].

The role of different oncometabolites is intensively studied in many tumourigenic events (pseudohypoxia, DNA methylation, histone modification etc.) and bioenergetics of tumour cells. Moreover, the expected regulatory role of 2-HG was also concluded in reprogramming of normal stromal cells, which could have tumour promoting effects on the microenvironment, as well [24]. *IDH1* mutations are described in 80% of gliomas, 20% of acute myeloid leukemias (AMLs) and in certain cholangiocarcinomas, thyroid cancers and chondrosarcomas [23, 25]. There are no data available about the role of mTOR activity in 2-HG production in any homozygous or heterozygous IDH mutant cells. Considering the aberrant regulatory impact of mTOR in malignant cells the question has been addressed whether mTORC1 operates through controlling of oncometabolite accumulation in metabolic reprogramming.

In the present work, a heterozygous *IDH1* mutant cell line and its rapamycin sensitivity were studied in vitro and in vivo. Our new results proved the role of mTOR activity and the inhibitory effect of rapamycin both in lactate and in 2-HG oncometabolite productions of heterozygous *IDH1* mutant fibrosarcoma cells.

## Methods

All materials were purchased from Sigma-Aldrich, except where it is indicated in the text.

### In vitro cell cultures and different treatments

HT-1080 endogenous heterozygous *IDH1* mutant cell line was used for both in vitro and in vivo experiments. HT-1080 (CCL121-ATCC); KMH2, DEV (human Hodgkin lymphoma cells purchased from DSMZ), ZR-75.1 (CRL-1500-ATCC), U251 MG homozygous *IDH1*-R132H mutant (kindly provided by prof. W. Leenders) [26]) cells were cultured in RPMI 1640 (R8758) or DMEM high glucose (Biosera, LM-D1108) (for U251 MG) medium supplemented with 10% (20% for DEV) fetal bovine serum (Hyclone) and gentamycin (for U251 MG penicillin-streptomycin). For different measurements, we plated the cells into T25 or T75 flasks (for Western blot, LC-MS and ^13^C-labelling experiments) or onto 6-, 96-well plates (for flow cytometry and Alamar Blue proliferation tests) using appropriate cell numbers (3-8×10^5^ cells/flask or 2-50×10^3^ cells/well). Cells were treated with rapamycin (0.01–200 ng/mL), PP242 (1 μM, Tocris), BPTES (10 μM), Zaprinast (1-100-300 μM), sodium-L-lactate (5 mM), sodium D-2-hydroxyglutarate (0.5 mM) for 24–72 h. In ^13^C-labelling metabolic experiments, the plated cultures were washed (with DMEM D5030 – glucose-, glutamine- and pyruvate-free medium) and the medium was replaced by DMEM D5030 medium during labelling.

For ^13^C-labelling cells were incubated with 10 mM U-^13^C-glucose or 4 mM U-^13^C-glutamine or 10 mM 2-^13^C-acetate (Cambridge Isotope Laboratories, Andover, MA, USA) in D5030 medium for one hour before the extraction.

### Cell proliferation, apoptosis and migration assays

To analyse the proliferation capacity Alamar Blue test was used (Thermo Fisher Scientific) – the incubation period was 4 h. Fluorescence was measured by Fluoroscan Ascent FL fluorimeter software (Labsystems International). Percentage of proliferation was given relative to control samples.

Apoptosis and cell cycle were measured by SubG1 analysis as described by Nagy et al. [27]; 10^6^ cells were fixed in ice-cold ethanol (70%), followed by alkaline extraction (200 mM Na_2_HPO_2_, pH 7.4), then stained with propidium-iodide. The measurements were carried out with FACSCalibur (BD Biosciences) and analysed by Kaluza software (Beckman Coulter).

Vertical migration from monolayer into extracellular matrix (ECM; MAXGel ECM) gel was studied using untreated and rapamycin treated (48 h, 50 ng/mL) HT-1080 cells (5×10^4^) in six-well micro-plates and then ECM was overlaid and the tumour cells were allowed to migrate into the gel in the next 24 h. At the end of the incubation period the ECM gel was removed from the monolayer, the cells were released from both compartments and were counted in hemocytometer. The migratory capacity was given in the percentage of the cells which migrated into the matrix after rapamycin treatment related to the controls [28].

### Expression analysis of different proteins by Western blot and flow cytometry

For Western blot analysis protein extracts from lysated cells were quantitated using Quant-iT protein assay (Invitrogen) and were separated by SDS-PAGE. Proteins were transferred to PVDF membrane applying semidry technique (BioRad), membranes were incubated with the following antibodies: anti-phospho-mTOR (Ser2448, 1:1000, #2971; Cell Signaling Technologies – CST), anti-phospho-S6 (Ser 235/236, 1:1000, #2211; CST), anti-Rictor (1:1000, #2140; CST), anti-Raptor (1:500, #89603; Novus), anti-mTOR (1:1000, #2938; CST), anti-S6 (1:1000, #2317; CST), anti-lactate dehydrogenase A (LDH-A) (1:1000; #3582, Cell Signaling), anti-glutaminase (Gls) (1:1000; #15676, Abcam) and anti-β-actin (1:5000; #A228, Sigma-Aldrich) as loading control. Finally, biotinylated secondary antibodies, avidin-HRP complex (Vectastain Elite ABC Kit, Vector) and enhanced chemiluminescence technique (Pierce ECL Western Blotting Substrate using Kodak-Xmat) were used.

For flow cytometric measurements 10^6^ cells were fixed and permeabilized using IntraStain Kit (DAKO), then were stained with the appropriate primary antibodies anti-LDH-A (1:200; #3582, Cell Signaling), anti-Gls (1:250; #15676, Abcam) after different treatments. LDH-A and Gls expressions were detected with secondary anti-rabbit IgG Alexa-Fluor 647 antibody (Invitrogen). Samples were measured by Navios flow cytometer (Beckman Coulter) and analysed by Kaluza software (Beckman Coulter). Displayed results are mean fluorescence intensities (MFI).

### Confirmation of IDH mutation by Sanger sequencing

Both *IDH1* and *IDH2* genes were analysed after using routine DNA Isolation kit for Cells and Tissue (Roche), specific amplifications (AmpliTaqGold Master Mix with the appropriate primers - *IDH1* exon4 forward: aaaactttgcttctaatttttctcttt; reverse: acatacaagttggaaatttctgg,; *IDH2* exon4 forward: tctagactctactgccttcctc; reverse: gtcagtggatcccctctcca – AppliedBiosystems), purification (ExoSAP-IT – Affimetrix) and direct sequencing (25 cycles at 51 °C, BigDye 3Terminator v3.1 Cycle Sequencing Kit in Genetic Analyser 3500 - Applied BioSystem).

### Metabolite analysis using liquid chromatography mass spectrometry

Intracellular metabolites (lactate, citrate, malate, succinate, 2-HG) were extracted by a modified method based on Szoboszlai et al. [29]. In brief, the cells were quenched in liquid nitrogen and extracted by mixture of MeOH–chloroform–H_2_O (9:1:1) and vortexed at 4 °C. After centrifugation (15,000xg, 10 min, 4 °C) the clear supernatants were kept at −80 °C. The samples were prepared for LC-MS by the established derivatization based on the protocol of Jaitz et al. [30]. For derivatization 3-nitrobenzyl-alcohol + trimethyl-chlorsilane were added to the dried samples, sonicated and incubated at 80 °C for 45 min. The reaction was stopped by 100 mM ammonium-hydrogencarbonate solution. After these processes the samples were diluted in acetonitrile-water solution. Gradient elution was used with reversed-phase chromatography in Waters Acquity LC system. The detection was performed by Waters Micromass Quattro Micro triple quadrupole mass spectrometer (Waters Corporation, Milford MA, USA) using electrospray source in the positive ion mode with single ion monitoring mode. Standards (L-lactic acid, L-malic acid, succinic acid, citric acid, D-2-hydroxyglutarate) and other chemicals except for labelled substrates were purchased from Sigma-Aldrich for these measurements. The analytes were detected in multiple reaction monitoring mode in MS. HT-1080 cells produce only D-2-HG [31]. Applying this method, we did not distinguish L- and D- 2-HG enantiomers and we use 2-HG, as a synonym for D-2-HG in the manuscript.

For ^13^C-labelling, cells were incubated with 10 mM U-^13^C-glucose or 4 mM U-^13^C- glutamine or 10 mM 2-^13^C-acetate (Cambridge Isotope Laboratories, Andover, MA, USA) in D5030 medium for one hour before the extraction.

### In vivo studies with HT-1080 xenograft model

Xenograft tumours were established in SCID mice by injecting 2 × 10^6^ HT-1080 cells subcutaneously (s.c.) into the sole region of 8–10-week-old (20–23 g) mice. Palpable tumours were removed and cut into equal pieces then were transplanted into secondary recipient mice. When palpable s.c. tumours developed, animals were divided into control and rapamycin-treated groups (*n* = 10 each). Rapamycin (Rapamune 1 mg/mL, Wyeth Europa Ltd.) was administered by gavage at 3 mg/kg body weight three times per week for 3 weeks. Control groups were treated with saline. Body weight and tumour size were determined. Tumour volume was calculated as follows: п/6 × (2 × shorter diameter + longer diameter)/3)^3^. Tumour weights were measured in euthanized animals at the end of the experiments. Tumour tissues were formalin-fixed and paraffin-embedded. Immunostaining was performed by anti-p-S6 (1:100; #2211; CST), anti-p-mTOR (1:100; 2971, CST), anti-Gls (1:200, #15676, Abcam) and anti-LDH-A (1:400; #3582, CST) antibodies according to Krencz et al [32]. Frozen tumour samples were also preserved at -80 °C for LC-MS metabolite extraction. In vivo experiments were approved by the Institutional Ethical Review Board (PEI/001/2457-6/2015) and the Institutional Animal Care Laboratory, with official permissions (PEI/001/1733-2/2015).

### Statistical analysis

The data are presented as mean ± standard deviation and calculated from three independent experiments with minimum three or more parallels, depending on the used method. Data evaluation was performed using Student’s *t (two-tailed)* test and variance analysis (ANOVA) for multiple comparisons with PAST 3.05 software. *p <* 0.05 was considered statistically significant.

## Results

### The source of elevated oncometabolite levels (lactate and 2-HG) in IDH1 mutant HT-1080 cells

**Fig. 1:**
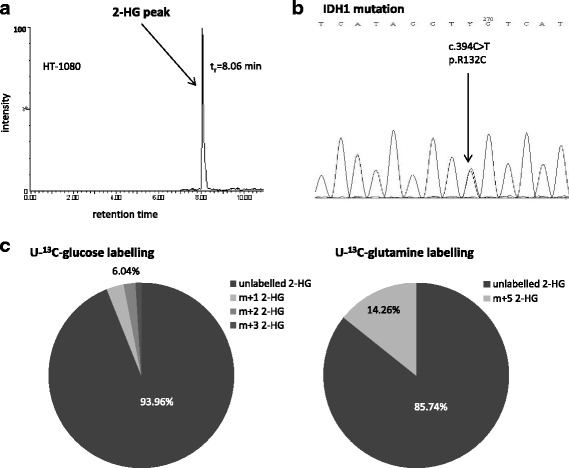
Consequence of *IDH1* mutation in HT-1080. The detected 2-HG peak on chromatogram (LC-MS) (**a**.) and the heterozygous *IDH1* gene mutation which resulted *IDH1* R132C was confirmed by sequencing (Sanger) (**b**.); The main sources of 2-HG production were evaluated by ^13^C incorporations in 2-HG after one-hour U-^13^C-glucose or U-^13^C-glutamine labelling and LC-MS (representative data) (**c**.). t_r_: retention time; m + 0 = unlabelled metabolites; m + 1/2/3 = 1/2/3 ^13^C in 2-HG from glucose, m + 5 = 5 ^13^C in 2-HG from glutamine, (m + 4 and m + 5 isotopomers were not detectable in these cells after one-hour glucose labelling in 2-HG)

Characterising the dominant bioenergetic process in HT-1080 fibrosarcoma cells increased glycolysis and impaired TCA (tricarboxylic acid) cycle were observed in our previous study [33]. 2-HG production (Fig. 1a) – high level of this oncometabolite was detected by LC-MS – and the decreased number and irregular arrangement of the cristae in mitochondria (electron microscopy-data not shown) represent other remarkable properties of HT-1080 cells. To elucidate the involvement of *IDH1* in the enhanced production of 2-HG the exons of *IDH1* gene were sequenced. Heterozygous *IDH1* gene mutation (R132C) was confirmed by Sanger sequencing in the studied HT-1080 cells (Fig. 1b).

High levels of lactate (~27 nmol/10^6^ cells) and 2-HG (~18 nmol/10^6^ cells) regarded as oncometabolites were characteristic features of HT-1080. The sources of the produced oncometabolites were studied after using different ^13^C labelled substrates (glucose, glutamine, acetate) and LC-MS. Incorporation of ^13^C atoms was shown in ~16% of lactate after one-hour ^13^C glucose labelling in HT-1080 cultures. However, neither U-^13^C glutamine nor 2-^13^C acetate could label lactate in these cells. Low level of ^13^C labelling from glucose was detected in 2-HG (~6% of total 2-HG pool), as well. Acetate substrate could not label 2-HG. However, glutamine was proved to be a remarkable source (~15%) of 2-HG (Fig. 1c).

### Rapamycin inhibited mTOR related protein expression, cell proliferation and migration of HT-1080 cells

**Fig. 2:**
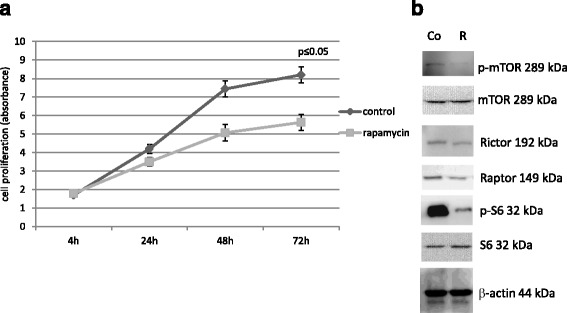
In vitro effects of rapamycin in HT-1080 cells (anti-proliferative effect and the decreased expressions of mTOR activity related proteins). The anti-proliferative effect of rapamycin was confirmed by Alamar Blue test after 4-24-48-72-h (the results of a representative experiment *n* = 6, *:*p* < 0.05) (**a**.). Reduced expressions of mTOR kinase activity related proteins (p-S6 and p-mTOR) were shown after 48-h rapamycin (50 ng/mL) treatment by Western blot analysis (representative blot) Co = control; R = rapamycin (**b**.)

The role of the described high mTORC1 activity [33] was studied in our work by rapamycin (mTORC1 inhibitor) treatment. Dose dependent anti-proliferative effect (0.1–200 ng/mL) of rapamycin was tested in vitro; based on the evaluation of our and other published results 50 ng/mL was selected for further in vitro studies. Both Alamar Blue test and the evaluation of cell number confirmed the time dependent effect (Fig. 2a) of 50 ng/mL rapamycin, as well. These were supported by flow cytometry, however, apoptotic effect was not detected after rapamycin treatment in these *IDH1* mutant cells after 24–72-h treatment (data not shown). The migration of HT-1080 cells was also inhibited after rapamycin treatment in ECM gels, the number of the migrated cells was reduced by more than 80%. Rapamycin sensitivity was confirmed after 48-h treatment by Western blot (Fig. 2b). Densitometric analysis showed significantly decreased expression in phosphorylated forms of S6 and mTOR (more than 85% decreasing in both p-S6 and p-mTOR, besides the total level of S6 and mTOR did not change). In parallel, Raptor and Rictor expressions were also reduced.

### Rapamycin decreased lactate and 2-HG oncometabolite production by reducing the amount of LDH-A and glutaminase enzymes

**Fig. 3:**
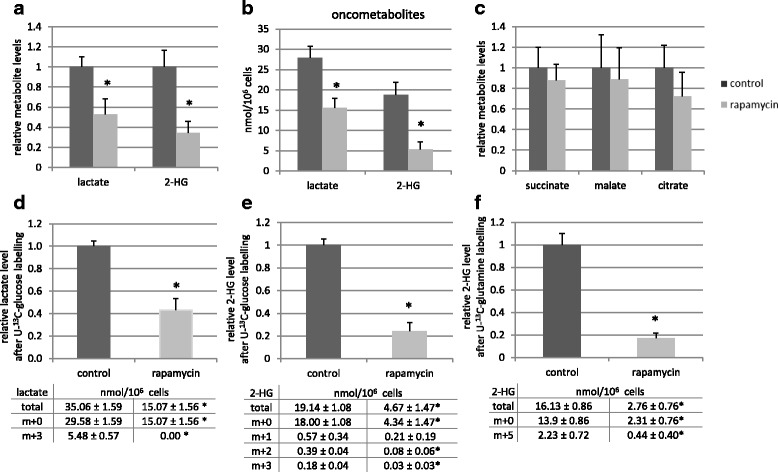
Rapamycin treatment reduced lactate and 2-HG productions in vitro (LC-MS measurements). The relative levels of lactate and 2-HG (**a**.); the levels of these oncometabolites in nmol/10^6^ cells (**b**.); the relative levels of succinate, malate and citrate (**c**.) after 48-h rapamycin treatment (50 ng/mL); The changes in relative level of total lactate (m + 0 and m + 3) were shown after 48-h rapamycin treatment and one-hour U-^13^C-glucose labelling (**d**.); The reduced level of total 2-HG (m + 0 and m + 1/2/3) was also detected after rapamycin treatment and U-^13^C-glucose labelling (**e**.) or U-^13^C-glutamine substrate labelling (**f**.) m + 0 = unlabelled metabolites; m + 3 = 3 ^13^C in lactate from glucose, m + 1/2/3 = 1/2/3 ^13^C in 2-HG from glucose, m + 5 = 5 ^13^C in 2-HG from glutamine, (m + 4 and m + 5 isotopomers were not detectable in these cells after one-hour glucose labelling in 2-HG); Levels of metabolites were expressed relative to the control (control level = 1) and the tables show the absolute levels of labelled and unlabelled metabolites after rapamycin treatment. (*n* = 3, *:*p* < 0.05;)

Significantly decreased intracellular levels of lactate and 2-HG were detected using LC-MS measurements after in vitro rapamycin treatment (Fig. 3a, b). Rapamycin altered the levels of various TCA cycle intermediates, as well; however, these changes were not proved to be significant (Fig. 3c). The effect of the remaining 2-HG and lactate levels in parallel with 48-72-h rapamycin treatment were also tested in vitro by adding 2-HG and/or lactate. In our experimental systems, we could not observe significant influences of lactate and/or 2-HG in proliferation, in growth potential and in rapamycin sensitivity of HT-1080 cells (Additional file 1: Figure S1.).

The metabolic effect was verified by using one-hour ^13^C-substrate labelling after 48-h rapamycin treatment and measuring-evaluating unlabelled and labelled metabolite levels by LC-MS. ^13^C atoms appeared in 16% of lactate from U-^13^C-glucose in control HT-1080 cells at these experimental conditions. 48-h rapamycin treatment reduced the level of total lactate (both ^12^C- and ^13^C-lactate) by more than 50% and blocked the incorporation of ^13^C from U-^13^C-glucose into lactate using one-hour labelling period at the end of the treatments (Fig. 3d). 2-HG production was also reduced effectively after rapamycin treatment, U-^13^C-glucose and U-^13^C-glutamine labelling studies confirmed that rapamycin inhibited 2-HG production. The total level of 2-HG was reduced by more than 75% and the amount of ^13^C-labelled 2-HG was also reduced significantly after using either glucose or glutamine labelling in one-hour time period in the treated cells compared to controls (Fig. 3e, f).

**Fig. 4:**
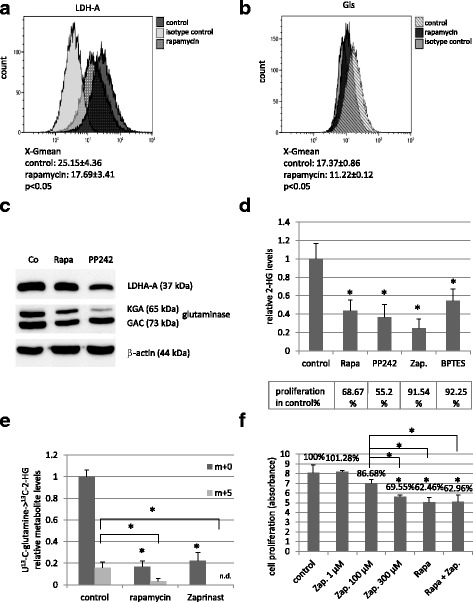
mTOR inhibitors (rapamycin, PP242) decreased the expressions of LDH-A and glutaminase proteins in HT-1080 cells; and glutaminase inhibitors (Zaprinast, BPTES) have effects on both the proliferation and the 2-HG oncometabolite level in these cells. LDH-A and Gls expressions were significantly reduced after 48 h rapamycin (50 ng/ml) and PP242 (1 μM) treatment (**a**. and **b**. show flow cytometric results, X-Gmean average ± SD n = 3,*:*p* < 0.05; **c**. Western blot). The effects of mTOR and glutaminase inhibitors on the 2-HG production and proliferation (**d**.) (Alamar Blue test; Rapa.: rapamycin 50 ng/mL; PP242: 1 μM; Zap.: Zaprinast 100 μM; BPTES: 10 μM). The effect of rapamycin and Zaprinast on the 2-HG production (**e**.) (LC-MS using U-^13^C-glutamine labelling; n = 3, levels of metabolites were expressed relative to the control (control level = 1), m + 0 = unlabelled 2-HG; m + 5 = 5 ^13^C in 2-HG, n.d.: not detected; *:*p* < 0.05). The results of Alamar Blue test after using 48-h Zaprinast and rapamycin (50 ng/ml) combination treatments in HT-1080 cells (**f**.)

The regulatory role of mTORC1 activity in both LDH-A and glutaminase expressions were analysed by flow cytometry in these experiments. Rapamycin reduced the expression of LDH-A and glutaminase at protein levels in HT-1080 cells after 48-h treatment (Fig. 4a, b). These were further confirmed by Western blot, as well (Fig. 4c). The role of glutaminase enzyme activity in 2-HG production was proved by Zaprinast and BPTES treatments in HT-1080 cells. Similar to rapamycin, Zaprinast and BPTES could also reduce 2-HG production (Fig. 4d). This was occurred at enzyme activity level [34], as glutaminase inhibition did not influence the protein expression of glutaminase negatively (data not shown). Zaprinast also reduced the proliferation of HT-1080 cells in vitro (Fig. 4e, f). However, rapamycin was significantly more effective than Zaprinast after 48–72-h treatment. Moreover, rapamycin and Zaprinast showed no additive effect on proliferation, this combination was not more effective than rapamycin alone (Fig. 4f).

To exclude non-target effect of rapamycin, as a chemical compound, we used an other mTOR inhibitor (PP242) and compared their effects on 2-HG, lactate production and on LDH-A, Gls protein expression and on proliferation, as well. PP242 is an ATP-competitive compound, which is highly selective to mTOR kinase in both mTORC1 and C2 complexes; it is a non-FKBP12 binding drug as mTORC1 inhibitor rapamycin. In addition, PP242 has similar effects on proliferation, Gls and LDH-A protein level (Fig. 4c, d), in parallel PP242 reduced the lactate level near to 60%, the 2-HG level near to 40%. These suggest that the inhibition of mTOR kinase is involved in the alteration of these parameters.

The lactate production of highly glycolytic other non-IDH mutant cell lines were also tested after in vitro rapamycin treatment in two human Hodgkin lymphoma cells and in another previously metabolically characterised ZR-75.1 human breast carcinoma cell line. 48–72 h rapamycin treatment could reduce the amount of lactate in these cells; the unlabelled lactate levels were significantly reduced to 85%–65% (DEV-75%, KMH2-85%, ZR-75.1–65%) in these other malignant cells. The use of one-hour U-^13^C-glucose labelling at the end of rapamycin treatments could confirm these changes, the labelled ^13^C-lactate level decreased to 30% in DEV and near to undetectable level in the other two cell lines in our experiments. Furthermore, we have chance to analyse 2-HG production of a generated (genetically modified) homozygous *IDH1* mutant glioma cell line – U251 MG. In these cells, we detected high 2-HG production (~24 nmol/10^6^ cells), however, the known low glycolytic capacity of this kind of glioma cells was also observed. The elevated 2-HG and proliferation levels were significantly reduced (near to 75% and 80%, respectively) after 48-h mTOR inhibitor treatment which confirmed our findings about the 2-HG reducing effect of rapamycin treatment in other 2-HG producing tumour type, in glioma cells.

### Rapamycin inhibited the growth and the oncometabolite production in HT-1080 xenografts in vivo

**Fig. 5:**
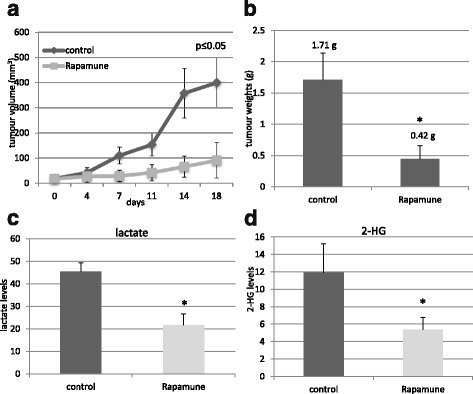
The effects of rapamycin on tumour growth and on oncometabolite production *in vivo*. Growth curve of the tumour (**a**.), and the measured tumour weights (**b**.), lactate (**c**.) and 2-HG (**d**.) levels of control and Rapamune (3 mg/kg, 3 weeks) treated HT-1080 xenografts (LC-MS, *n* = 6,*:*p* < 0.05; metabolite levels in nmol/10 mg tumour mass were given

The in vivo inhibitory effect of mTORC1 inhibitor Rapamune (oral rapamycin drug) on tumour growth was also confirmed in HT-1080 xenotransplanted SCID mice. Rapamune treatment significantly reduced tumour weights and tumour volumes in vivo (Fig. 5a, b). The in vivo inhibitory effect of the applied treatment on mTORC1 activity and on the expression of Gls and LDH-A were confirmed by using p-S6, p-mTOR, Gls and LDH-A immunostainings (Additional file 2: Figure S2.). The significantly reduced oncometabolite levels – lactate and 2-HG – were also detected by LC-MS in frozen tumour tissues of HT-1080 xenografts (Fig. 5c, d).

## Discussion

Several studies have shown that metabolic intermediates have potential to modulate oncogenic (including cellular and extracellular mechanisms) and epigenetic processes. Moreover, the accumulation of certain metabolites with normal or altered structures – designated as oncometabolites – may contribute to carcinogenesis in many aspects [35, 36].

**Fig. 6:**
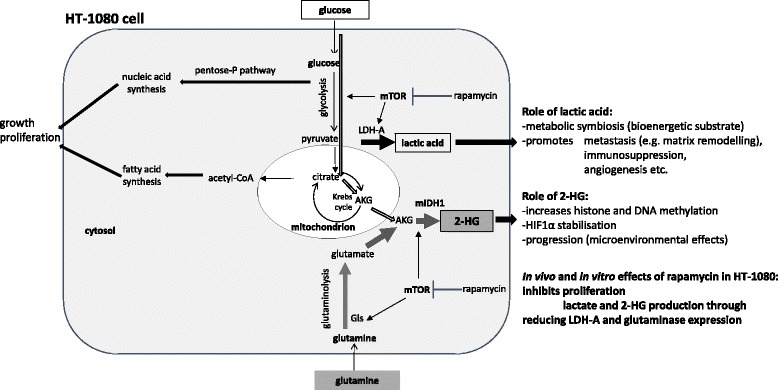
The effect of rapamycin on metabolic profile of *IDH1* mutant HT-1080 cells. Rapamycin treatment reduced the levels of two oncometabolites in parallel - lactate and 2-HG - in high glycolytic, *IDH1* mutant fibrosarcoma cells. mTOR inhibition decreased metabolic capacity, the glycolysis and the glutaminolysis with remarkable downregulation of LDH-A and glutaminase enzyme expressions in HT-1080 cells (further explanation in the discussion)

Noteworthy, oncometabolites are interconnected and form a network to act by cooperative or autoregulated manner in tumourigenesis, as well [35–38]. Recently, Kottakis et al. have supported this network concept especially at epigenetic level in correlation with the functions of mTOR controlled pathways [39]. This work underlines the importance of our new data about mTOR activity dependent regulation of lactate and 2-HG productions in m*IDH1* HT-1080 fibrosarcoma. The presented data showed that mTOR complex inhibitors – in parallel reduce tumour growth and migration – have significant inhibitory effect on the productions of both glycolytic lactate and mainly from glutamine sourced 2-HG oncometabolites in vitro and in vivo. According to our results and the known microenvironmental tumour promoting effects of tumour derived lactate, not only the well-known anti-proliferative effect of mTOR inhibitors should be considered. The altered levels of oncometabolites, especially their potential extracellular effects should also be taken into account in case of starting to use rapalog treatments. For example, doxorubicin inhibits the proliferation, however, it could not significantly alter the studied oncometabolite levels in HT-1080 cells (data not shown, unpublished results). The reduction of lactate and 2-HG oncometabolites could have fewer effects on stable tumour cell lines in short-term treatments than in their microenvironment and in the associated inflammatory or other “normal” (non-tumour) cells at tissue level. The reduced levels of oncometabolites are interesting for further studies because rapamycin preferentially reduced the productions of lactate and 2-HG relative to succinate, malate and citrate. Our observations call the attention to the translational effects of mTOR activity [37, 40], which were proved by the detected decrease of LDH-A and Gls expressions at protein level both in vitro and in vivo (Fig. 6.).

As far as the regulatory role of mTOR in protein translation is concerned, there are only limited data about mTOR hyperactivity dependent metabolic alterations related to oncometabolite productions, especially 2-HG. The detected correlation between hyperactivated mTOR and LDH expressions and the related lactate production corresponds to the known regulatory role of mTOR activity in the expressions of glycolytic enzymes [41, 42]. Various interactions between mTOR activity and metabolic alterations including oncometabolite production have been studied in few available reports applying different experimental conditions. It has been reported that 2-HG could equally activate and inhibit mTOR in different cells (genetically manipulated glioblastoma, *C. elegans* etc.) [43, 44]. Since different biological systems have been used in these experiments, there is a possibility that the outcome of 2-HG action has been influenced by different modulators such as origin and timing of their presence. However, these antagonistic impacts between oncometabolites and mTOR activity may suggest a possible coordination of lactate and 2-HG for joint tumour promoting functions. Nevertheless, it is noteworthy that our conclusion based on the obtained results – with an established primarily heterozygous mutant *IDH1* fibrosarcoma cell line – are in harmony with those reported by Carbonneau et al [44]. Their results have demonstrated mTOR kinase activation in other models in different tumour cells (HeLa, immortalized astrocytes) after octyl-2-HG treatment or in newly mIDH transfected cells.

Herein we confirmed their findings that mIDH can lead both to 2-HG production and to aberrant activation of mTOR kinase [44] using other primarily endogenous heterozygous *IDH1* mutant cell line. However, our new findings provide that the hyperactivity of mTOR kinase is needed to produce 2-HG in the studied primarily *IDH1* mutant cells.

Nevertheless, the presented data support our view that the detected reductions at different oncometabolite levels correlate to the inhibited proliferation, migration after rapamycin treatment in HT-1080 cells in vitro. The detected decrease of mTOR activity and its correlation with diminished oncometabolite level and tumour growth were also demonstrated in vitro xenografts in our study. This effect of rapamycin treatment highlights the importance of mTOR activity in malignant phenotype of *IDH1* mutant cells. Our results – concerning the potential roles of mTOR activity and 2-HG production in migration of malignant cells – confirm the recently published data about the enhanced migration capacity of mIDH1 transfected glioma cells [45]. Only this study and our work show the potential benefit of mTOR inhibitors on the migration of IDH mutation related malignant cellular phenotype, in this context.

The anti-tumour effects of rapalogs, other mTOR inhibitors and elevated mTOR activity have been described previously in many tumours including gliomas, AMLs and chondrosarcomas (where the occurrence of the IDH mutation is remarkable) [46]. Rapalogs and newly developed mTOR inhibitors are being tested with recurrent and therapy resistant malignancies, especially in AML and glioma patients [47–49].

Based on our recent findings and the emerging data about the relation between high mTOR activity and IDH mutation, we also highlight the importance of recently ongoing and future studies in different AMLs, gliomas, and sarcomas. We also suggest studying the metabolic profile, IDH status or oncometabolite productions – e.g. lactate and 2-HG level – in these patients. Our results showed that the endogenous mutant *IDH1* expressing HT-1080 fibrosarcoma cells with glycolytic phenotype proved to be a suitable model to characterise the role of mTOR in the regulation of 2-HG and lactate productions. The present study provided evidence for the first time for simultaneous reduction of two oncometabolites (lactate and 2-HG) by mTOR inhibitor. Moreover, we could detect such a reducing effect of rapamycin in oncometabolite level, especially in glycolytic lactate production in other lymphoma and breast cancer cells.

Other laboratories – with separate studies on either lactate or 2-HG expression– have described that specific glycolysis or glutaminolysis inhibitors (such as deoxyglucose or Zaprinast, BPTES) can reduce the cellular proliferation in vitro, however, they have no significant efficacy in vivo in monotherapy [34, 50, 51]. The mTOR inhibitors (rapamycin mTORC1 and PP242 C1-C2 inhibitors) showed high efficacy in our study. Rapamycin and PP242 significantly inhibited tumour growth in HT-1080 IDH mutant cells, where the productions of two oncometabolites were also attenuated. High concentrations of lactate and 2-HG exert tumour promoting potential in a cooperative manner with the surveillance of mTOR activity (e.g. 2-HG initiates DNA hypermethylation and lactate fuels the bioenergetic process) [16, 52] which can be present in the studied HT-1080 cells. Certainly, the above outlined network and its role in the potential metabolic symbiosis of the tissue microenvironment could be regarded as an important phenomenon. However, this cannot be denoted as an exclusive mechanism to maintain malignant phenotype of HT-1080 fibrosarcoma cells. In addition, it has been published recently by E. Allen et al. that inhibition of mTOR in combination can disrupt the metabolic symbiosis of normoxic and hypoxic cells in the developing targeted therapy resistance [53]. This and our new results suggest that targeting mTOR activity could have a promising potential in different new combination treatments depending on the metabolic phenotype of tumours.

These and our recent findings highlight that tumour promoting mechanisms supported jointly by lactate and 2-HG oncometabolites – regulated by mTOR dependent translational effects –, and especially the role of metabolic crossroads would be a promising approach to recognise a set of molecular targets for anticancer therapy.

## Conclusions

In conclusion, the mTORC1 inhibitor (rapamycin) showed high efficacy in our models. The high mTOR activity related biological advances in tumour proliferation were inhibited by rapamycin both in vitro and in vivo in the studied heterozygous *IDH1* mutant cells. Moreover, the altered metabolic profile, the consequences of metabolic adaptation to *IDH1* mutation and to other tumourigenic alterations (e.g. hyperactivated glycolytic pathway) such as the high levels of two oncometabolites – 2-HG and lactate – were also disrupted after the specific effect of rapamycin treatment. Considering the role of these oncometabolites in intracellular and microenvironmental regulatory networks and their role in metabolic symbiosis it could be regarded that rapamycin and other developing mTOR inhibitors may have additional effects besides their anti-proliferative effects in different tumours with characteristic highly glycolytic phenotype, especially in case of *IDH1* mutation (such as AML, gliomas, chondrosarcomas). Based on our new results we could suggest targeting mTOR activity depending on the metabolic besides molecular genetic phenotype of tumours to increase the success of the future therapies.

## Additional files

Additional file 1: Figure S1. Adding 2-HG, lactate did not influence the anti-proliferative effect of rapamycin treatment in HT-1080 cells. The in vitro anti-proliferative effect of rapamycin (50 ng/ml), Na-L-lactate (5 mM) and D-2-HG (0.5 mM) combination treatments were studied by Alamar Blue test after 48–72 h (the results of a representative experiment *n* = 6, *:*p* < 0.05; Rapa = rapamycin, 2-HG = 2-hydroxyglutarate, lac = lactate) (PDF 147 kb).

Additional file 2: Figure S2. The representative photos of p-mTOR, p-S6, LDH-A and Gls immunostainings in HT-1080 xenograft tumours. The expressions of p-mTOR, p-S6, LDH-A and Gls were studied in control and Rapamune treated xenograft tumours (DAB-brown staining; magnification 400X) (PDF 209 kb).

## Abbreviations

2-HG: 2-hydroxyglutarate
4-EBP1: 4E-binding protein
Akt: v-akt murine thymoma viral oncogene homolog
AML: Acute myeloid leukemia
FH: Fumarate hydratase
Gls: Glutaminase
IDH: Isocitrate dehydrogenase
LDH: Lactate dehydrogenase
mIDH: Mutant isocitrate dehydrogenase
mTOR: Mammalian target of rapamycin
p70S6K: Phospho70S6 kinase
PI3K: Phosphatidilinositol-3-kinase
PKC: Protein kinase C
SDH: Succinate dehydrogenase
SGK1: Serum/glucocorticoid regulated kinase 1
TCA: Tricarboxylic acid.

## References

[1] Laplante M, Sabatini DM (2012). mTOR signaling in growth control and disease. Cell.

[2] Shimobayashi M, Hall MN (2014). Making new contacts: the mTOR network in metabolism and signalling crosstalk. Nat Rev Mol Cell Biol.

[3] Albert V, Hall MN (2015). mTOR signaling in cellular and organismal energetics. Curr Opin Cell Biol.

[4] Zoncu R, Efeyan A, Sabatini DM (2011). MTOR: from growth signal integration to cancer, diabetes and ageing. Nat Rev Mol Cell Biol.

[5] Dienstmann R, Rodon J, Serra V, Tabernero J (2014). Picking the point of inhibition: a comparative review of PI3K/AKT/mTOR pathway inhibitors. Mol Cancer Ther.

[6] Sticz T, Molnár A, Márk Á, Hajdu M, Nagy N, Végső G, et al. mTOR activity and its prognostic significance in human colorectal carcinoma depending on C1 and C2 complex-related protein expression. J Clin Pathol 2016 doi:10.1136/jclinpath-2016-203913. 10.1136/jclinpath-2016-203913 27729429

[7] Bian Y, Wang Z, Xu J, Zhao W, Cao H, Zhang Z (2015). Elevated Rictor expression is associated with tumor progression and poor prognosis in patients with gastric cancer. Biochem Biophys Res Commun.

[8] Wildey G, Behtaj M, Kresak A, Yang M, Fu P, Dowlati A (2016). RICTOR amplification identifies a subgroup in small cell lung cancer and predicts response to drugs targeting mTOR. Oncotarget.

[9] Makinoshima H, Takita M, Saruwatari K, Umemura S, Obata Y, Ishii G (2015). Signaling through the Phosphatidylinositol 3-Kinase (PI3K)/Mammalian Target of Rapamycin (mTOR) Axis Is Responsible for Aerobic Glycolysis mediated by Glucose Transporter in Epidermal Growth Factor Receptor (EGFR)-mutated Lung Adenocarcinoma. J Biol Chem.

[10] Yu L, Chen X, Wang L, Chen S (2016). The sweet trap in tumors: aerobic glycolysis and potential targets for therapy. Oncotarget.

[11] Villar VH, Merhi F, Djavaheri-Mergny M, Durán RV (2015). Glutaminolysis and autophagy in cancer. Autophagy.

[12] Pusapati RV, Daemen A, Wilson C, Sandoval W, Gao M, Haley B (2016). mTORC1-Dependent Metabolic Reprogramming Underlies Escape from Glycolysis Addiction in Cancer Cells. Cancer Cell.

[13] Tanaka K, Sasayama T, Irino Y, Takata K, Nagashima H, Satoh N (2015). Compensatory glutamine metabolism promotes glioblastoma resistance to mTOR inhibitor treatment. J Clin Invest.

[14] Li J, Shin S, Sun Y, Yoon SO, Li C, Zhang E (2016). mTORC1-Driven Tumor Cells Are Highly Sensitive to Therapeutic Targeting by Antagonists of Oxidative Stress. Cancer Res.

[15] Zha X, Sun Q, Zhang H (2011). mTOR upregulation of glycolytic enzymes promotes tumor development. Cell Cycle.

[16] Van Hée VF, Pérez-Escuredo J, Cacace A, Copetti T, Sonveaux P (2015). Lactate does not activate NF-κB in oxidative tumor cells. Front Pharmacol.

[17] Nowicki S, Gottlieb E (2015). Oncometabolites: tailoring our genes. FEBS J.

[18] Morin A, Letouzé E, Gimenez-Roqueplo AP, Favier J (2014). Oncometabolites-driven tumorigenesis: From genetics to targeted therapy. Int J Cancer.

[19] Payen VL, Porporato PE, Baselet B, Sonveaux P (2016). Metabolic changes associated with tumor metastasis, part 1: tumor pH, glycolysis and the pentose phosphate pathway. Cell Mol Life Sci.

[20] Marchiq I, Pouysségur J (2016). Hypoxia, cancer metabolism and the therapeutic benefit of targeting lactate/H^+^ symporters. J Mol Med (Berl).

[21] Yoshida GJ (2015). Metabolic reprogramming: the emerging concept and associated therapeutic strategies. J Exp Clin Cancer Res.

[22] Oermann EK, Wu J, Guan KL, Xiong Y (2012). Alterations of metabolic genes and metabolites in cancer. Semin Cell Dev Biol.

[23] Clark O, Yen K, Mellinghoff IK (2016). Molecular Pathways: Isocitrate Dehydrogenase Mutations in Cancer. Clin Cancer Res.

[24] Chen JY, Lai YS, Tsai HJ, Kuo CC, Yen BL, Yeh SP (2016). The oncometabolite R-2 hydroxyglutarate activates NF-κB-dependent tumor-promoting stromal niche for acute myeloid leukemia cells. Sci Rep..

[25] Waitkus MS, Diplas BH, Yan H (2016). Isocitrate dehydrogenase mutations in gliomas. Neuro-Oncology.

[26] Esmaeili M, Hamans BC, Navis AC, van Horssen R, Bathen TF, Gribbestad IS, Leenders WP, Heerschap A (2014). IDH1 R132H mutation generates a distinct phospholipid metabolite profile in glioma. Cancer Res.

[27] Nagy N, Hajdu M, Márk Á, Király PA, Tóth M, Dankó T (2016). Growth inhibitory effect of rapamycin in Hodgkin-lymphoma cell lines characterized by constitutive NOTCH1 activation. Tumour Biol.

[28] Pogány G, Timár F, Oláh J, Harisi R, Polony G, Paku S (2001). Role of the basement membrane in tumor cell dormancy and cytotoxic resistance. Oncology.

[29] Szoboszlai N, Guo X, Ozohanics O, Oláh J, Gömöry Á, Mihucz VG (2014). Determination of energy metabolites in cancer cells by porous graphitic carbon liquid chromatography electrospray ionization mass spectrometry for the assessment of energy metabolism. Anal Chim Acta.

[30] Jaitz L, Mueller B, Koellensperger G, Huber D, Oburger E, Puschenreiter M (2011). LC-MS analysis of low molecular weight organic acids derived from root exudation. Anal Bioanal Chem.

[31] Li L, Paz AC, Wilky BA, Johnson B, Galoian K, Rosenberg A (2015). Treatment with a Small Molecule Mutant IDH1 Inhibitor Suppresses Tumorigenic Activity and Decreases Production of the Oncometabolite 2-Hydroxyglutarate in Human Chondrosarcoma Cells. PLoS One.

[32] Krencz I, Sebestyén A, Fábián K, Márk Á, Moldvay J, Khoor A, et al. Expression of mTORC1/2-related proteins in primary and brain metastatic lung adenocarcinoma. Hum Pathol 2016 doi:10.1016/j.humpath.2016.12.012. (Epub ahead of print). 10.1016/j.humpath.2016.12.012 28025080

[33] Jeney A, Hujber Z, Szoboszlai N, Fullár A, Oláh J, Pap É (2016). Characterisation of bioenergetic pathways and related regulators by multiple assays in human tumour cells. Cancer Cell Int.

[34] Elhammali A, Ippolito JE, Collins L, Crowley J, Marasa J, Piwnica-Worms D (2014). A high-throughput fluorimetric assay for 2-hydroxyglutarate identifies Zaprinast as a glutaminase inhibitor. Cancer Discov.

[35] Gao X, Locasale JW (2016). Serine metabolism links tumor suppression to the epigenetic landscape. Cell Metab.

[36] Colvin H, Nishida N, Konno M, Haraguchi N, Takahashi H, Nishimura J (2016). Oncometabolite D-2-Hydroxyglurate directly induces epithelial-mesenchymal transition and is associated with distant metastasis in colorectal cancer. Sci Rep.

[37] Sciacovelli M, Gonçalves E, Johnson TI, Zecchini VR, da Costa AS, Gaude E (2016). Fumarate is an epigenetic modifier that elicits epithelial-to-mesenchymal transition. Nature.

[38] Li F, He X, Ye D, Lin Y, Yu H, Yao C (2015). NADP(+)-IDH Mutations Promote Hypersuccinylation that Impairs Mitochondria Respiration and Induces Apoptosis Resistance. Mol Cell.

[39] Kottakis F, Nicolay BN, Roumane A, Karnik R, Gu H, Nagle JM (2016). LKB1 loss links serine metabolism to DNA methylation and tumorigenesis. Nature.

[40] Li J, Kim SG, Blenis J (2014). Rapamycin: one drug, many effects. Cell Metab.

[41] Chen X, Zhu Y, Wang Z, Zhu H, Pan Q, Su S (2016). mTORC1 alters the expression of glycolytic genes by regulating KPNA2 abundances. J Proteomics.

[42] Düvel K, Yecies JL, Menon S, Raman P, Lipovsky AI, Souza AL (2010). Activation of a metabolic gene regulatory network downstream of mTOR complex 1. Mol Cell.

[43] Fu X, Chin RM, Vergnes L, Hwang H, Deng G, Xing Y (2015). 2-Hydroxyglutarate Inhibits ATP Synthase and mTOR Signaling. Cell Metab.

[44] Carbonneau M, M Gagné L, Lalonde ME, Germain MA, Motorina A, Guiot MC (2016). 2-hydroxyglutarate activates the mTOR signalling pathway. Nat Commun.

[45] Zhu H, Zhang Y, Chen J, Qiu J, Huang K, Wu M (2017). IDH1 R132H Mutation Enhances Cell Migration by Activating AKT-mTOR Signaling Pathway, but Sensitizes Cells to 5-FU Treatment as NADPH and GSH Are Reduced. PLoS One.

[46] Tan P, Soo Tiong I, Fleming S, Pomilio G, Cummings N, Droogleever M (2016). The mTOR inhibitor everolimus in combination with azacitidine in patients with relapsed/refractory acute myeloid leukemia: a phase Ib/II study. Oncotarget.

[47] Brenner AK, Andersson Tvedt TH, Bruserud Ø. The Complexity of Targeting PI3K-Akt-mTOR Signalling in Human Acute Myeloid Leukaemia: The Importance of Leukemic Cell Heterogeneity, Neighbouring Mesenchymal Stem Cells and Immunocompetent Cells. Molecules 2016;21(11). 10.3390/molecules21111512 PMC6273124 27845732

[48] Miller JJ, Wen PY (2016). Emerging targeted therapies for glioma. Expert Opin Emerg Drugs.

[49] Becher OJ, Gilheeney SW, Khakoo Y, Lyden DC, Haque S, De Braganca KC, et al. A phase I study of perifosine with temsirolimus for recurrent pediatric solid tumors. Pediatr Blood Cancer 2016 doi: 10.1002/pbc.26409. (Epub ahead of print). 10.1002/pbc.26409 28035748

[50] Zhao X, Jiang P, Deng X, Li Z, Tian F, Guo F (2016). Inhibition of mTORC1 signaling sensitizes hepatocellular carcinoma cells to glycolytic stress. Am J Cancer Res.

[51] Chen L, Cui H, Fang J, Deng H, Kuang P, Guo H (2016). Glutamine deprivation plus BPTES alters etoposide- and cisplatin-induced apoptosis in triple negative breast cancer cells. Oncotarget.

[52] Jin Y, Elalaf H, Watanabe M, Tamaki S, Hineno S, Matsunaga K (2015). Mutant IDH1 Dysregulates the Differentiation of Mesenchymal Stem Cells in Association with Gene-Specific Histone Modifications to Cartilage- and Bone-Related Genes. PLoS One.

[53] Allen E, Miéville P, Warren CM, Saghafinia S, Li L, Peng MW (2016). Metabolic Symbiosis Enables Adaptive Resistance to Anti-angiogenic Therapy that Is Dependent on mTOR Signaling. Cell Rep.

