# Supplementary figures and images for: Rapamycin (mTORC1 inhibitor) reduces the production of lactate and 2-hydroxyglutarate oncometabolites in IDH1 mutant fibrosarcoma cells
Source: J Exp Clin Cancer Res. 2017 Jun 2;36:74. doi: 10.1186/s13046-017-0544-y (PMC5457553; doi:10.1186/s13046-017-0544-y)

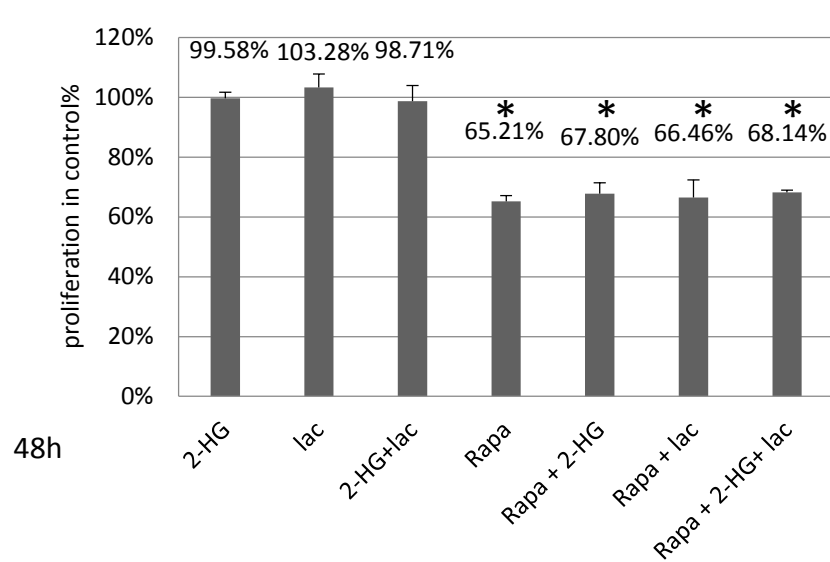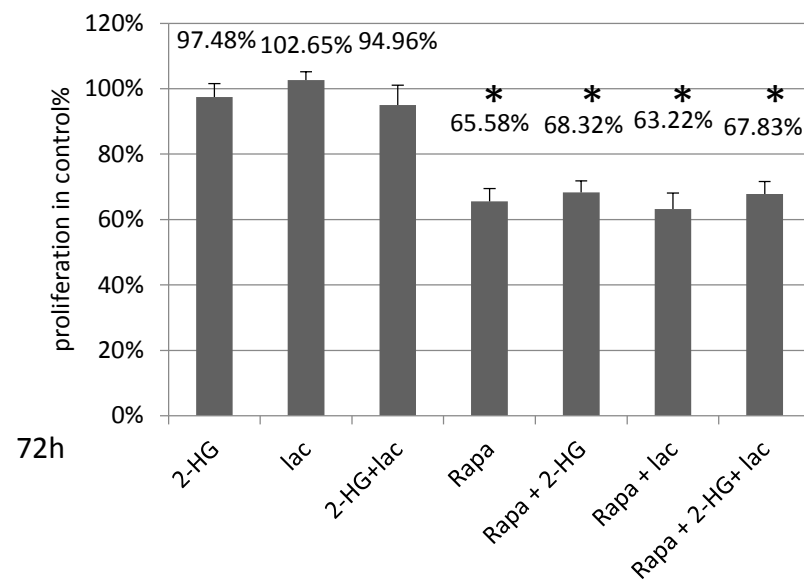

Suppl. Figure 1.

Supplement: Supplementary file 1 — Adding 2-HG, lactate did not influence the anti-proliferative effect of rapamycin treatment in HT-1080 cells. The in vitro anti-proliferative effect of rapamycin (50 ng/ml), Na-L-lactate (5 mM) and D-2-HG (0.5 mM) combination treatments were studied by Alamar Blue test after 48–72 h (the results of a representative experiment n = 6, *:p < 0.05; Rapa = rapamycin, 2-HG = 2-hydroxyglutarate, lac = lactate) (PDF 147 kb). [file 13046_2017_544_MOESM1_ESM.pdf]

p-mTOR

p-S6

LDH-A

Gls

control

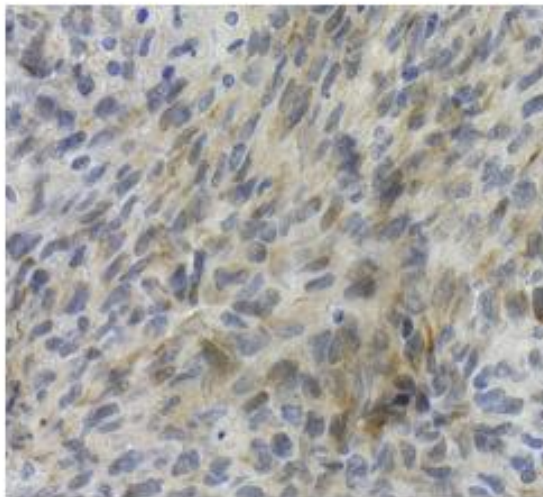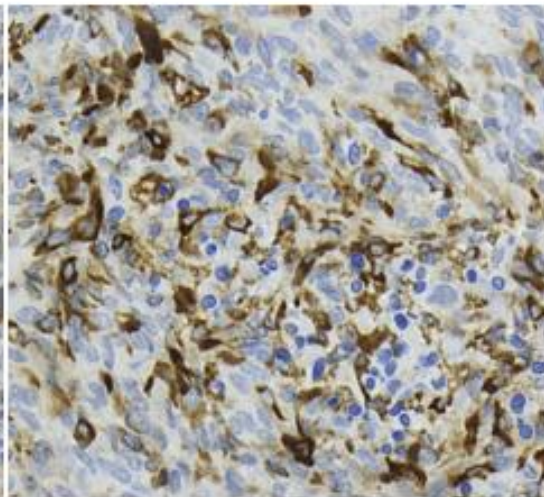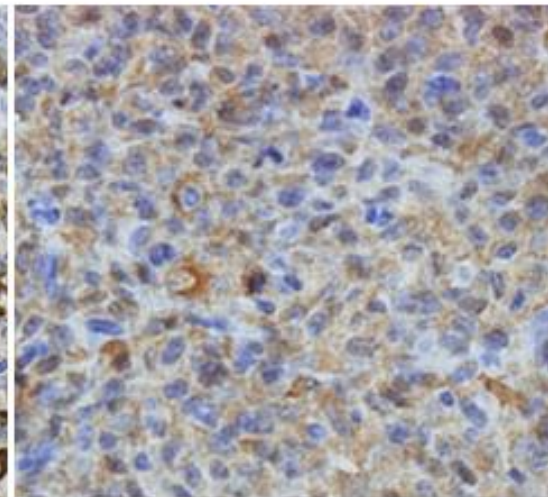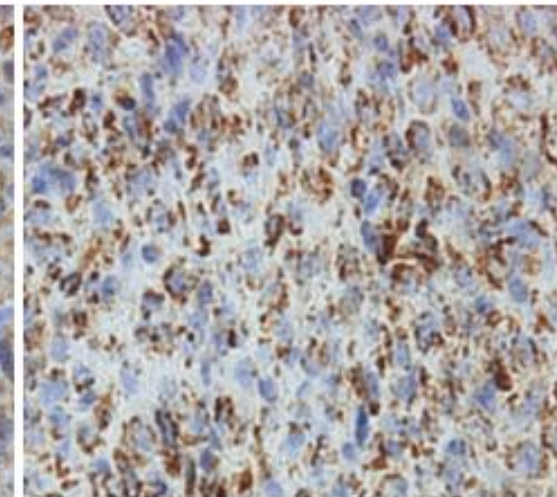

Rapamune

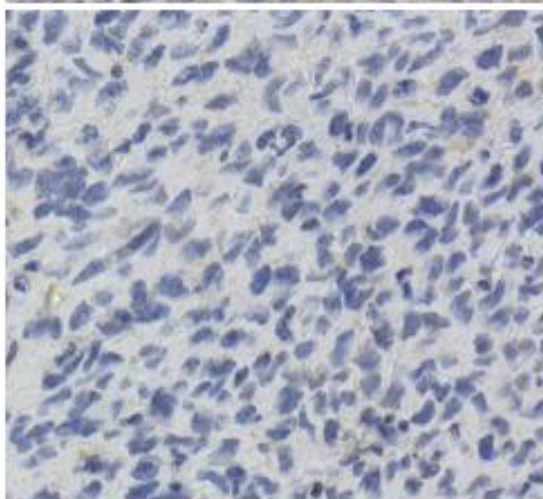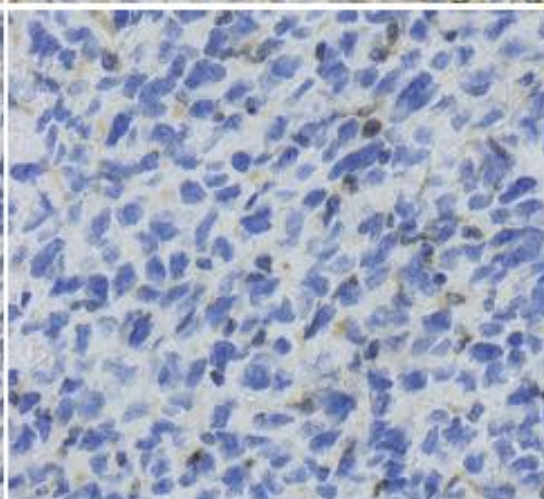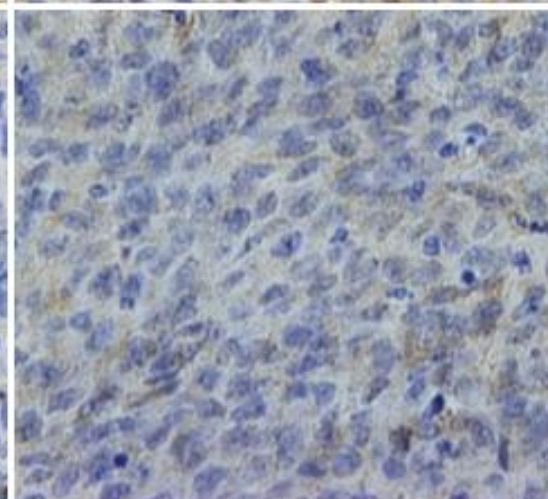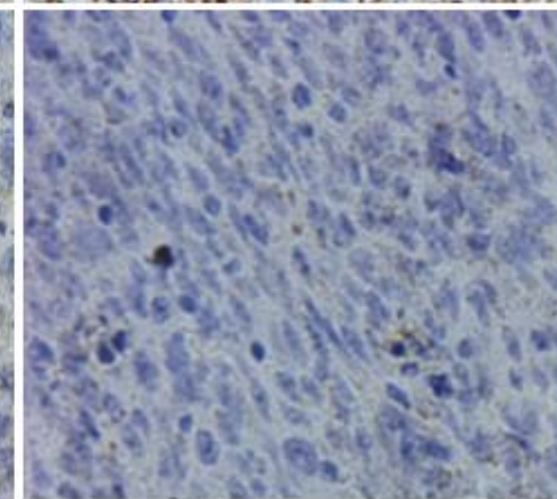

Suppl. Figure 1.

Supplement: Supplementary file 2 — The representative photos of p-mTOR, p-S6, LDH-A and Gls immunostainings in HT-1080 xenograft tumours. The expressions of p-mTOR, p-S6, LDH-A and Gls were studied in control and Rapamune treated xenograft tumours (DAB-brown staining; magnification 400X) (PDF 209 kb). [file 13046_2017_544_MOESM2_ESM.pdf]
